# Crosstalk between Macrophages, T Cells, and Iron Metabolism in Tumor Microenvironment

**DOI:** 10.1155/2021/8865791

**Published:** 2021-02-02

**Authors:** Lesang Shen, Yunxiang Zhou, Haifei He, Wuzhen Chen, Cameron Lenahan, Xiaoyi Li, Yongchuan Deng, Anwen Shao, Jian Huang

**Affiliations:** ^1^Department of Breast Surgery, The Second Affiliated Hospital, School of Medicine, Zhejiang University, Hangzhou 310009, China; ^2^Key Laboratory of Tumor Microenvironment and Immune Therapy of Zhejiang Province, Hangzhou 310009, China; ^3^Department of Surgical Oncology, The Second Affiliated Hospital, School of Medicine, Zhejiang University, Hangzhou 310009, China; ^4^Burrell College of Osteopathic Medicine, Las Cruces, NM, USA; ^5^Department of Nuclear Medicine and PET-CT Center, The Second Affiliated Hospital, Zhejiang University, Hangzhou 310009, China; ^6^Department of Neurosurgery, The Second Affiliated Hospital, School of Medicine, Zhejiang University, Hangzhou 310009, China

## Abstract

Leukocytes, including macrophages and T cells, represent key players in the human immune system, which plays a considerable role in the development and progression of tumors by immune surveillance or immune escape. Boosting the recruitment of leukocytes into the tumor microenvironment and promoting their antitumor responses have been hot areas of research in recent years. Although immunotherapy has manifested a certain level of success in some malignancies, the overall effectiveness is far from satisfactory. Iron is an essential trace element required in multiple, normal cellular processes, such as DNA synthesis and repair, cellular respiration, metabolism, and signaling, while dysregulated iron metabolism has been declared one of the metabolic hallmarks of malignant cancer cells. Furthermore, iron is implicated in the modulation of innate and adaptive immune responses, and elucidating the targeted regulation of iron metabolism may have the potential to benefit antitumor immunity and cancer treatment. In the present review, we briefly summarize the roles of leukocytes and iron metabolism in tumorigenesis, as well as their crosstalk in the tumor microenvironment. The combination of immunotherapy with targeted regulation of iron and iron-dependent regulated cell death (ferroptosis) may be a focus of future research.

## 1. Introduction

Cancer incidence and mortality are rapidly growing around the world, with an estimated 18 million new diagnosed cases and 9.6 million cancer-related deaths in 2018[1]. Cancer still represents a large social and economic burden in each country despite increased public awareness of cancer-related lifestyle factors and applications of early screening and diagnosis [2]. The past decade has witnessed substantial progress in the areas of tumor genomics and biology and technologies in cancer research. Two newly proposed cancer hallmarks, tumor-associated inflammation and tumor immune evasion, have highlighted the close interaction between the immune system and cancer [3, 4]. The tumor microenvironment (TME) is composed of cancer cells, stromal cells, extracellular matrix, and immune cells, which influence tumorigenesis, tumor expansion, and metastasis [5]. Recruited leukocytes paradoxically inhibit or promote tumor initiation and progression, depending on the cytokines and chemokines that are secreted by the TME, as well as the type and stage of the tumor. This phenomenon is known as tumor immunoediting, which comprises the dual tumor-suppressing and tumor-promoting actions of immunity [6, 7]. There is a great need to understand the complex crosstalk among immune cells, cancer cells, and the TME and to develop innovative therapeutic strategies for the treatment of cancer.

Iron is an essential trace element required in normal cellular processes, including DNA synthesis and repair, cellular respiration, metabolism, and signaling [8]. Dysregulation of iron metabolism has been implicated in several diseases, such as anemia [9], infections [10], neurodegenerative disorders [11], and cancer [12]. The capacity of iron to undergo redox reactions enables iron to catalyze the Fenton reaction that generates reactive oxygen species (ROS). The consequence may induce tumorigenesis through DNA damage, as well as lipid and protein modifications in neoplastic cells [12, 13]. There is also emerging evidence proving the role of iron in tumor development, metastasis, and TME modification [13, 14]. In this review, we briefly introduce the pathophysiology of iron metabolism and leukocytes, especially macrophages and T cells. We also summarize the current knowledge regarding their representative role, as well as their crosstalk in the tumor microenvironment, providing a better understanding of the underlying mechanisms of tumorigenesis and offering novel insight into cancer therapy.

## 2. Tumor Immune Surveillance

The human immune system is a dynamic and intricate network, which is responsible for the defense of the host body against attacks by harmful substances, including its own cells when they become malignant. Tumor progression, from development to metastasis, and its response to therapy are intimately influenced by the activity of the immune system [15].

### 2.1. Overview of Immune Surveillance

The first line of defense is innate immunity, and the cells involved are macrophages, neutrophils, dendritic cells (DCs), and natural killer (NK) cells [16]. If pathogens succeed in avoiding innate defenses, a more versatile and intricate adaptive immune response is triggered, which is mediated by B lymphocytes and T lymphocytes [16]. These two levels of immunity are distinct, but they are interacting components that collectively protect against pathogens and foreign proteins [17].

In recent years, the exciting progress achieved in tumor immunotherapy, such as adoptive T cell therapies and immune checkpoint inhibitors, has attracted much more attention in this field [18]. In the 1950s, the concept of “cancer immunosurveillance” was proposed by Burnet [19], which is based on the notion that the expression of tumor-associated antigens and tumor-specific antigens (neoantigens) induces antitumor immunity, leading to the destruction of transformed and/or malignant cells before early neoplasms develop into detectable cancers [20]. A series of steps must be initiated to generate an antitumor immune response, elegantly summarized in the process, known as the “cancer-immunity cycle” [21], which includes the capture of tumor antigens by specialized antigen presenting cells (APCs), the priming and activation of T cells, and the trafficking and infiltration of cytotoxic T lymphocytes (CTLs) to the tumor. Eventually, cytotoxic immune cells, such as CTLs and NK cells, attack and eliminate cancer cells with high immunogenicity [22].

### 2.2. Leukocyte Recruitment and Function within the Tumor Microenvironment

Since 1863, when Virchow first observed the presence of leukocytes within tumors, an association between inflammation and cancer has gradually become a hot topic in cancer research [23, 24]. Tumor-associated inflammation is now accepted as one of the hallmarks of cancer, which contributes to genomic instability, epigenetic change, cancer cell proliferation, angiogenesis, invasion, and metastasis [3]. For example, chronic inflammation influenced by hepatitis B and C viruses increases the risk for liver cancer, and infections with *Helicobacter pylori* favor the development of gastric cancer [25]. Additionally, non-steroidal anti-inflammatory drugs, such as aspirin, have been demonstrated to reduce the incidence and mortality in many types of cancer, contributing to their nonspecific, inhibitory effects on inflammation [26, 27].

Leukocytic recruitment into tumors relies on the local production of chemoattractants (e.g., chemokines) and their cognate receptors expressed by leukocytes [28]. Infiltrated inflammatory immune cells, cancer cells, and a network of stromal cells that are comprised of fibroblasts and endothelial cells constitute dominant components of the TME. The cross-talk between cancer cells and nonneoplastic cells is believed to shape and regulate tumor development, partially through signaling molecules, including growth factors, chemokines, cytokines, and exosomes [29, 30]. With the development of genetic manipulation technology and pharmacological inhibitors, the anticancer or oncogenic functions of tumor-infiltrating immune cells have been highlighted [3, 23]. For example, NK cells are a major source of tumor necrosis factor-*α* (TNF-*α*), interferon-gamma (INF-*γ*), and granulocyte-macrophage colony stimulating factor (GM-CSF), and they can initiate antitumor effects mainly by secreting cytotoxic perforin and granzyme when stimulatory cell face receptors, such as NKG2D, are bound [31]. DCs also play a significant role in antitumor immunity by expressing cytokines and chemokines to promote the priming of antitumor T cells, including interleukin-1 (IL-1), IL-12, and CXC-chemokine ligand 9 (CXCL9) [32]. Moreover, infiltration of effector T lymphocytes in tumors [helper 1 (Th1) cells and CTLs] is associated with prolonged patient survival in most solid cancers [33]. Meanwhile, other immunosuppressive cells, such as myeloid-derived suppressor cells, T regulatory cells (Tregs), and tumor-associated macrophages (TAMs), participate in facilitating immune escape and sustaining tumor growth [15, 29], which will be described in detail below.

Taken together, anticancer immunity exerts immunosurveillance of tumor immunogenicity, while tumor-associated inflammation promotes tumor progression by suppressing antitumor immunity in the TME and by providing direct pro-tumorigenic signaling onto epithelial and cancer cells [30]. The intricate relationship between them is dependent upon the time and context of the tumorigenesis.

## 3. Tumor Immune Escape

**Figure 1:**
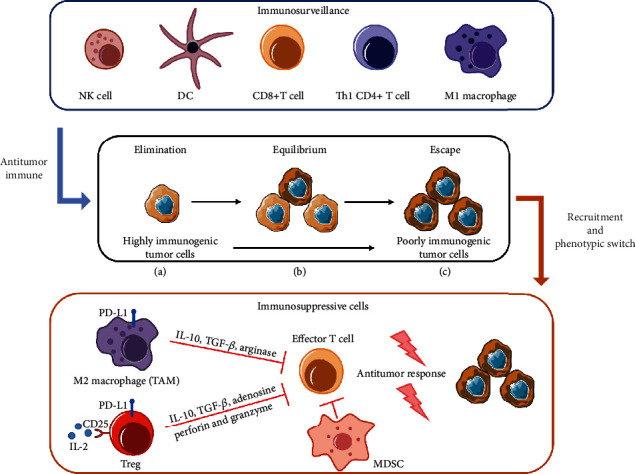
The procedure of tumor immunoediting: from immune surveillance to immune escape. During the elimination phase, the immunosurveillance components of both innate and adaptive immune systems recognize and eliminate tumor cells. Tumors gradually acquire immune-escape capability in the setting of chronic inflammation and immune dysregulation. Eventually, cancer cells secrete cytokines and chemokines to recruit immunosuppressive cells, including TAMs, Tregs, and MDSCs, which suppress antitumor immune responses through different pathways. DC: dendritic cell; IL: interleukin; MDSC: myeloid-derived suppressor cell; NK: natural killer; PD-L1: programmed cell death-ligand 1; TGF: transforming growth factor; Th: helper T; Treg: T regulatory cell.

Tumors grow progressively out of immune control after “tumor immunoediting” (Figure 1), a process comprising three phases (elimination, equilibrium, and escape) [7, 34]. Escape from immunosurveillance inevitably leads to the progression of cancer and failure of immunotherapy. There are several mechanisms of tumor immune escape known in the TME, including impaired antigen presentation, poor immunogenicity of cancer cells, and the absence of costimulatory molecules that induce tolerance of T cells [35]. More importantly, a number of immunosuppressive cells (e.g., TAMs and Tregs) are induced and recruited by tumors, producing a favorable environment for cancer cells [36, 37]. Because these immunosuppressive cells are considered targets for immunotherapy resistance, it is necessary to elucidate how they contribute to tumor immune escape.

### 3.1. Tumor-Associated Macrophages in Immune Escape

Macrophages are differentiated cells of the mononuclear phagocyte system and are components of the immune system found within many tissues, where they play a key role in anti-infective immunity, wound repair, and tissue homeostasis [38]. Circulating monocytes are recruited to the tumors and become TAMs by a multitude of chemokines, including CCL2, CCL5, CXCL12, and colony-stimulating factor-1 (CSF-1), and by the presence of local hypoxia and high levels of lactic acid in the TME [39, 40]. As the largest inflammatory fraction in most human solid malignancies, TAMs are involved in all stages of tumor progression, from carcinoma cell proliferation throughout dissemination to metastasis [29]. Consequently, increased TAM infiltration generally correlates with poor prognosis, but not always, in animal models and oncological patients [39, 41].

TAM activation is complicated and comprises two extreme states: M1 macrophages [proinflammatory phenotype, driven by IFN-*γ*, lipopolysaccharide, or GM-CSF] and M2 macrophages (anti-inflammatory phenotype, driven by IL-4, IL-13, or CSF-1), which is determined by the surrounding microenvironment [42]. Early in tumor development, M1-polarized macrophages are potent effector cells that are able to elicit tumor cell disruption. Conversely, as tumorigenesis progresses, the TME favors the transition of infiltrated macrophages to the M2 phenotype with protumorigenic activities [43]. TAMs are widely considered to be M2 macrophages, in that they promote tumor angiogenesis, cancer progression, and immunosuppression [44]. Accordingly, M2-like TAMs inhibit cytotoxic CD8+ T cell antitumor activity and DC maturation by the secretion of transforming growth factor-*β* (TGF-*β*) and IL-10[45, 46]. Furthermore, the inhibitor ligand programmed cell death-ligand 1 (PD-L1) is upregulated, not only in malignant cells but also in TAMs in response to IFN-*γ* from effector cells [47], and the interaction of PD-L1 with programmed cell death protein 1 (PD-1) expressed on activated T cells facilitates immune escape. TAMs also express PD-1, and the expression is inversely proportional to the phagocytic potency of TAMs, suggesting that the immune checkpoint inhibitor may be effective on macrophages [48]. From the immunometabolic perspective, M2-like TAMs deplete amino acids and secrete lactate among the TME, resulting in functional impairment of effector NK and T cells [49]. For these reasons, TAM can be a valid target for tumor immunotherapy, and several strategies controlling TAM function and polarization are emerging.

### 3.2. T Cells in Immune Escape

As the second greatest immune cell type besides TAMs in the TME, T cells are divided into two main categories: MHC-I restricted CD8+ T (cytotoxic T) cells and MHC-II restricted CD4+ T (helper T/Th) cells. Among them, CD8+ T cells differentiate into CTLs and exert a direct antitumor effect by releasing cytotoxic perforin, while Th1 cells mediate the antitumor response through the secretion of various cytokines, such as IFN-*γ*, TNF-*α*, and IL-2 to coordinate CTLs and NK cells [50]. Then, what are the immunosuppressive mechanisms that enable cancer cells to evade T cell attack?

The full activity of effector T cells depends on the antigenic peptide presented by APCs, as well as the engagement of costimulatory receptor, CD28 [51]. The two well-known coinhibitory molecules, PD-1 and cytotoxic T lymphocyte-associated antigen 4 (CTLA-4), play a major role in the maintenance of immune tolerance and in tumor immune evasion [18, 52]. In addition, the new generation of immune checkpoints, such as T cell immunoglobulin and mucin domain 3 (TIM-3) [53], lymphocyte activation gene 3 (LAG-3) [54], and V domain Ig suppressor of T cell activation (VISTA) [55], represents promising therapeutic targets for tumor immunotherapy.

Apart from engaging immune checkpoints, the TME and tumor cells block the antitumor immune response through the recruitment of Tregs. Tregs are defined as Foxp3+CD25+CD4+ T cells and function to suppress immunological and autoimmune diseases [56]. Previous studies have found that a high number of Tregs infiltrate into human tumor tissues and that the increase in tumor-infiltrating Tregs and a lower ratio of CD8+ cells to Treg cells in the TME are often correlated with an unfavorable prognosis [57, 58]. However, some exceptions exist, such as in colorectal cancer [59]. Compared with naïve Tregs, tumor-infiltrating Tregs highly express cell-surface molecules, such as CCR8, CD25 (IL-2 receptor), CTLA-4, PD-1, PD-L1, TIM-3, LAG-3, and VISTA [60]. There are various mechanisms of Treg-mediated immune suppression: (a) production of inhibitory cytokines, such as IL-10 and TGF-*β* [61]; (b) direct killing of effector T cells using perforin and granzyme [62]; (c) dominant consumption of IL-2 through high-affinity CD25[63]; and (d) expression of negative costimulatory molecules, such as CTLA-4 on the Treg surface, resulting in down-regulation of CD80/86 expression in APCs, therefore inhibiting T cell activation [64].

In summary, the immunosuppressive cells generate immunosuppressive cytokines and induce the emergence of immunosuppressive networks within the TME, promoting the evasion of antitumor immunity and supporting tumor progression.

## 4. Relationship between Iron Metabolism and Cancer

### 4.1. Cellular Iron Metabolism and Homeostasis

Multiple iron metabolism-associated molecules collaborate to maintain homeostasis because iron is a necessary, but potentially toxic, element [65]. Iron homeostasis is thus a strictly regulated process that involves uptake, storage, and utilization. Uptake of dietary iron is through divalent metal transporter 1 (DMT1) expressed on duodenum enterocytes. Then, iron exportation is mediated by ferroportin (FPN). Circulating iron predominantly binds to transferrin (TF), forming a complex named TF-bound iron (TBI), which recognizes transferrin receptor 1 (TFR1) [8]. Along with internalization of this complex by endocytosis, iron is reduced by six-transmembrane epithelial antigen of the prostate 3 (STEAP3) within the endosome and is subsequently released into the cytosol through DMT1 to constitute the cytoplasmic labile iron pool (LIP). The fate of this redox-active iron is to be stored in the form of ferritin (FT), utilized for various metabolic needs, or exported out of cells by FPN [66]. Finally, iron gets oxidized by ceruloplasmin or hephaestin and again combines with TF. In this regard, iron homeostasis at the cellular level is regulated by posttranscriptional mechanisms of iron-responsive element-binding proteins 1 and 2, which interact with iron responsive elements in response to levels of intracellular iron [67]. At the systemic level, iron homeostasis is primarily maintained by hepcidin, an important iron regulatory hormone. Under high-iron conditions, hepcidin is released by the liver and induces FPN degradation, preventing iron export from duodenum enterocytes, hepatocytes, and macrophages into the blood stream [68, 69]. In humans, mutations in the genes encoding hemochromatosis protein (HFE) and hemojuvelin (HJV) have been reported to cause low expression of hepcidin, which implies that HFE and HJV are key regulators of hepcidin [70]. Besides regulating iron metabolism, HFE is considered a nonclassical major histocompatibility complex- (MHC-) Ib molecule, playing an immunological role in impairing MHC-I antigen presentation and T cell activation [71]. The negative modulatory function of HFE has been identified in innate immunity against viral and bacterial infections [72, 73]. Furthermore, HJV mediates innate antimicrobial immune response via macrophages in the acute infection phase without relying on iron burden [74]. These observations suggest the immunological functions of iron-related genes.

### 4.2. Altered Iron Metabolism in Cancer

Dysregulated iron homeostasis is considered one of the metabolic hallmarks of malignant cancer cells, in which some pivotal alterations of iron import-export, storage, and regulation have been identified [8, 75]. These changes contribute to elevated levels of intracellular iron, which is critical in various pathophysiological processes, including cell cycle regulation, DNA synthesis, tumor development, metastasis, and TME modification [13, 14].

Upregulation of TFR1 is one way to increase iron uptake. It has been repeatedly observed that TFR1 is overexpressed in several cancers, including lung, ovarian, and breast cancer, as well as leukemia and glioblastoma [76]. In non-small-cell lung cancer, an epidermal growth factor receptor was found to modulate iron metabolism by binding to and regulating the subcellular distribution of TFR1 [77]. Increases in TFR1 are also directly driven by the proto-oncogene, c-Myc [78]. Conversely, knockdown of TFR1 is associated with reduced ROS and insufficient mitochondrial respiration in human pancreatic cancer cells. TFR1 expression determines the sensitivity of tumor cells to oxidative stress [79]. Another mechanism of intracellular iron accumulation in tumorigenesis is overexpressed DMT1, which is responsible for ferrous iron entry. Colon-specific DMT1 blocking has been shown to reduce tumor proliferation by suppressing JAK-STAT3 signaling in mouse colorectal cancer models [80]. Moreover, SLC39A14 [Zrt/Irt-like protein 14 (ZIP14)] [81] and SLC39A8 [Zrt/Irt-like protein 8 (ZIP8)] [82], first identified as zinc transporters, are also involved in non-TBI uptake. In human liver hepatoma cell lines, knockdown of p53 accelerated iron uptake by increasing ZIP14 levels, indicating the potential role of ZIP14 in p53-related cancers [83]. Most studies on ZIP8 have focused on its effects of zinc homeostasis, and its iron-dependent role in cancers remains largely unclear [84]. As known, ferrireductases, particularly members of the STEAP family, are involved in iron reduction for cellular uptake [85]. Multiple studies have indicated that STEAP1 and STEAP2 are upregulated in different human cancer tumors, such as cancers of the prostate, ovarian, pancreas, and bladder [86, 87]. In glioblastoma, expression levels of STEAP3 correlate negatively with patient prognosis, and STEAP3 mediates cancer progression through induction of epithelial-mesenchymal transition, promotion of TFR expression, and activation of STAT3-FoxM1 signaling [88].

Ferritin, composing ferritin heavy chains (FTH) and ferritin light chains (FTL), plays a central role in iron storage. Among cancer patients, high concentrations of plasma FT correlate with a higher tumor stage and poor clinical outcome, suggesting that FT can serve as a prognostic factor in some types of cancer, such as colorectal cancer, hepatobiliary cancer, prostate cancer, or squamous cell carcinoma [89–92]. Human breast cancer cells with a more aggressive mesenchymal phenotype exhibit higher levels of both FTL and FTH compared to cells with an epithelial phenotype [93]. Moreover, downregulation of FT accounts for increased chemosensitivity [93, 94], as well as inhibition of tumor growth and development [95, 96].

The iron export system is controlled by the only known iron exporter, FPN, and its modulator, hepcidin [68]. The expression levels of FPN are substantially reduced in prostate and breast cancer compared to those in normal tissues, and they correlate with the degree of tumor aggressiveness [97, 98]. Shan et al. demonstrated that suppressing FPN expression in triple negative breast cancer cells results in epithelial-mesenchymal transition, cell proliferation, and migration [99]. Pinnix et al. showed that FPN-transfected breast cancer cells markedly reduce their growth in orthotopic tumor models. Furthermore, through gene expression profile analysis of over 800 breast cancer patients, the group confirmed that reduced FPN levels are independently associated with a significant decrease in patient progression-free survival [98]. There is also much evidence indicating that patients with various cancers have elevated serum and tumor hepcidin levels [100, 101]. Hepcidin can be synthesized in cancer cells, functioning as an autocrine hormone to degrade membrane FPN, increase intracellular concentration, and promote tumor survival, a process jointly controlled by bone morphogenetic protein and IL-6 [102, 103]. Thus, targeting the hepcidin-FPN axis to reduce iron availability may be promising strategies with antitumor efficacy [104].

## 5. The Crosstalk between Iron Metabolism and Immune System in Tumor

**Figure 2:**
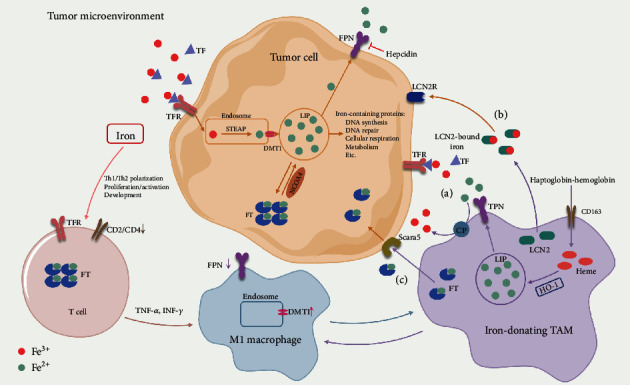
Overview of the iron-interplay among tumor cells, T cells, and macrophages in the tumor microenvironment. The anti-inflammatory TAMs adopt an iron-donating phenotype. TAMs rely on uptake of haptoglobin-hemoglobin complex through CD163 and on the expression of HO-1 to release heme-recycled iron into LIP. TAMs supply iron to accelerate tumor growth through three major pathways: (a) iron is exported via FPN and gets oxidized by CP and is then bound to TF to be taken up by tumor cells via TFR. (b) TAMs secrete LCN2, and then LCN2-bound iron is taken up by LCN2R on tumor cell surface. (c) TAM-released FT might be taken up by Scara5 (ferritin light chain binding protein). During tumor progression, iron metabolism-related proteins are aberrantly modified, such as upregulated TFR and DMT1, overexpressed FT, and dysregulated hepcidin-FPN axis. Moreover, iron is involved in T cell activation, Th1/Th2 polarization, and the downregulated surface expression of CD2/CD4. T cells secrete TNF-*α* and IFN-*γ*, which reduce FPN levels, but increase DMT1 levels, thus promoting iron-sequestering M1 macrophage polarization. CP: ceruloplasmin; DMT1: divalent metal transporter 1; FPN: ferroportin; FT: ferritin; HO-1: heme oxygenase-1; INF-*γ*: interferon-*γ*; LCN2: lipocalin-2; LCN2R: LCN2 receptor; LIP: labile iron pool; NCOA4: nuclear receptor coactivator 4; STEAP: six-transmembrane epithelial antigen of the prostate; TAM: tumor-associated macrophage; TF: transferrin; TFR: transferrin receptor; Th: helper T; TNF-*α*: tumor necrosis factor-*α*.

The essential role of iron in tumor development is tightly related to its ability to regulate innate and adaptive responses, especially in macrophages and T cells (Figure 2(a)–2(c)). Iron is undoubtedly required for immune cells to adapt their phenotype and acquire capacities for defense against pathogens or tumor cells regarded as foreign substances. It is therefore not surprising that immune cells compete with tumor cells for iron uptake in the TME. Meanwhile, immune cells modify the polarization state to modulate iron metabolism at both local tumor and systemic levels [105].

### 5.1. Iron Metabolism and Macrophages in Tumor

Macrophages have a central role in regulating systemic iron metabolism. During phagocytosis of senescent erythrocytes by macrophages in the spleen and the liver, heme is released and catabolized by heme oxygenase (mainly HO-1) to produce iron. This heme-recycled iron represents the majority of available iron in the body, then stored in FT or delivered to FPN [106]. Notably, the differential expression of iron-regulated genes is described among distinct macrophage phenotypes, indicating that macrophage polarization is associated with changes in iron homeostasis [107, 108]. In the early stages of tumorigenesis, proinflammatory cytokines promote M1-like macrophages (with low levels of FPN and high levels of FT) to display an iron sequestering phenotype as an antitumor response [108, 109]. In contrast, M2-like macrophages exhibit an iron-release phenotype with higher expression of the iron exporter, FPN, and lower expression of the storage protein, FT, thus increasing iron recycling and export into the extracellular space.

TAMs have been widely accepted as an anti-inflammatory “iron-donating” phenotype that releases iron to support cancer progression [110, 111]. They show a high expression of CD163, the high-affinity scavenger receptor that binds haptoglobin and hemoglobin into a complex for uptake [108]. Shiraishi et al. observed that macrophage-mediated tumor proliferation was abrogated by silencing CD163 in mice and human sarcoma cell lines, verifying the role of CD163 in protumor activation of macrophages [112]. Scavenging heme is closely related to HO-1 expression, and released heme induces the degradation of the transcription factor, BACH1, that in turn induces HO-1 and enhances the expression of FPN [113, 114]. The heme-recycled iron primarily enters the LIP rather than being stored in FT as seen in M1 macrophages, preferentially for the release into the local microenvironment [106]. Besides upregulation of FPN, *in vitro*, TAMs supply tumor cells with iron through the secreted iron-binding protein lipocalin-2 (LCN-2) [115, 116]. For example, in breast cancer, increased FPN1 expression of infiltrating macrophages coexists with overexpression of hepcidin at the same stage of carcinogenesis [111], and FPN depletion in TAM does not affect its iron-release or protumorigenic capacity[116]. Mechanistically, the secretion of LCN-2 is associated with apoptotic tumor cell-released sphingosine 1-phosphate, which binds to its coupled receptor and triggers STAT3 activation [117]. Furthermore, macrophages are able to secrete FT into the microenvironment to stimulate tumorigenesis, though this proliferative effect is possibly iron-independent [118]. Interestingly, in mouse lung carcinoma models, the TAM subpopulation in hemorrhagic regions shows an iron-loaded, proinflammatory phenotype capable of eliminating tumor cells [119]. It has been confirmed that such differentiation in the TME is driven by the heme and iron, which is ingested from damaged red blood cells through leaky tumor vessels. Of clinical significance, administration of iron nanoparticles is associated with M1 polarization, as well as tumor suppression function both *in vitro* and *in vivo* [119]. Considering the great heterogeneity and functional plasticity, as well as the central role of TAMs in regulating iron homeostasis within the TME, further research is warranted to clarify how iron influences the crosstalk between macrophages and tumor cells.

### 5.2. Iron Metabolism and T Cells in Tumor

Iron homeostasis is an important determinant of valid T cell-mediated immune response, as either iron overload or iron deficiency adversely impacts the adaptive immune response in human disease states [120, 121]. On one hand, T cells require iron for their proliferation and effector functions in the course of immune responses, such as infections or tumors. Activation of T cells is accompanied by the upregulation of TFR (also called CD71) through an IL2-dependent pathway, even in early T cell differentiation [122–124]. Conversely, the induction of T cell energy (defect in proliferative responses, which is undesirable in tumor) is concomitant with reduced TFR surface expression in mouse models [125]. A mutation in TFRC, the gene that encodes TFR1, was discovered in patients with a combined immunodeficiency, which results in impaired iron endocytosis and defective T and B cell function [126]. In addition, deletion of the FTH gene in hematopoietic cells reduces the quantity of T and B cells as a result of an increase in LIP and enlarged ROS formation, suggesting that the iron stored in FT is required for lymphocyte survival [127]. On the other hand, iron dyshomeostasis has been implicated in the abnormal proportion of T cell subpopulations. For example, patients with iron overload secondary to *β*-thalassemia have decreased CD4+ and increased CD8+ T cells [128], while patients suffering from HFE-associated hereditary hemochromatosis (HH) show altered CD4/CD8 ratios and reduced CTLs, depending on the HLA haplotype [129, 130]. Moreover, HH patients present with increased levels of IL-4 and IL-10 produced by CD8+ T cells, which may promote Th2 polarization in some cases, again relating to impaired cancer immune surveillance [131]. As expected, the cancer risk is relatively high in patients with HFE gene mutations [132]. Iron can also directly modulate T cell phenotypes and has been demonstrated to downregulate the surface expression of CD2 and CD4 *in vitro* [133]. In situations of iron-uptake suppression via inhibition of TFR or employment of iron chelators, Th1 cells seem more sensitive and more easily susceptible to intracellular iron depletion than Th2 cells [134, 135]. Hence, T cell function and iron metabolism are intimately related, but to what extent iron imbalances in the TME lead to immune defects and promote tumor progression requires in-depth exploration.

## 6. Regulation of Iron Metabolism for Antitumor Immunity

The immune contexture is now increasingly acknowledged as a major determinant of survival outcomes in patients with cancer, and effective antitumor immune responses are important for immune defense against tumors [33]. Although immunotherapy has manifested a certain level of success in some malignancies, the overall effectiveness is far from satisfactory because of de novo or adaptive resistance [136]. Therefore, additional strategies are required to improve therapeutic efficacy. Considering the significant role of iron in the progression of cancer and in the modulation of innate and adaptive immune responses, targeted regulation of iron metabolism may be beneficial for antitumor immunity and cancer treatment.

Iron chelation therapies were initially employed for iron overload diseases, such as *β*-thalassemia, before it was demonstrated that they had antitumor activity in both *in vitro* and *in vivo* studies [137]. For example, the Food and Drug Administration- (FDA-) approved iron chelators, deferoxamine and deferasirox, are proven to be effective in preclinical studies of leukemia [138, 139], neuroblastomas [140], and colorectal [141], pancreatic [142], and breast [143] cancers. Additionally, the more high-affinity chelator thiosemicarbazones (3-AP, triapine) have been evaluated in several phase I and II clinical trials specifically for treating cancer and have shown relatively favorable efficacy as a monotherapy or in combination therapies [144–146]. However, these agents are not yet approved for clinical cancer treatment, partly due to the serious dose-limiting toxicities, a lack of selectivity for targeting tumor cells, and unreached effective intratumor concentrations limited by unfavorable pharmacokinetics [105]. Interestingly, recent studies have noticed that the intracellular iron chelator, (TC3-S)_2_, can alter iron-donor phenotype of TAMs towards iron sequestering phenotype, with the former supporting tumor progression and metastasis [147]. Thus, iron chelators influence not only tumor cells but also other components within the TME, and macrophage-targeted chelation may potentially provide new therapeutic avenues for iron chelation treatment.

Over the last decade, iron oxide nanoparticles (IONPs), as an extensively utilized inorganic nanomaterial, have shown great efficacy in a variety of biomedical applications, including cancer diagnosis and treatment [148]. Recently, multiple studies have indicated that IONPs impact macrophage polarization and thus possess antitumor properties [119, 149, 150]. The FDA-approved iron nanoparticle, ferumoxytol, has been observed to induce apoptosis in mammary cancer cells coincubated with macrophages and prevent against the growth and metastases of lung and liver cancers in mice models [149]. Mechanistically, the intrinsic inhibitory effect of ferumoxytol is dependent on polarizing TAMs to the tumor-suppressing M1 phenotype that induces Fenton reactions [149]. Meanwhile, Li et al. confirmed that hyaluronic acid-decorated IONP- (HION-) reprogrammed macrophages display improved antitumor capabilities by generating ROS and proinflammatory cytokines in the immunosuppressive TME, even promoting intratumoral M2-to-M1 switch in a paracrine-like manner [151]. Furthermore, newly synthesized nanocomposites, IONP-ovalbumin (OVA) and iron oxide-embedded large-pore mesoporous organosilica nanosphere- (IO-LPMON-) OVA, could not only potentially polarize macrophages but could also induce DC-primed cytotoxic T cell activation to prevent tumor formation, showing excellent potential of combinatorial immunotherapy approaches [152, 153]. A recent study shed light on the mechanism that IONPs moderate the activation of the interferon regulatory factor 5 pathway for M1 polarization, a process that relies on intracellular iron levels and impairs M2 function by downregulating arginase-1 [150].

Ferroptosis is a form of iron-dependent regulated cell death characterized by the accumulation of lipid peroxidation [154]. The initiation of ferroptosis requires three indispensable hallmarks: the oxidation of phospholipids containing polyunsaturated fatty acid, the presence of intracellular free iron and iron-containing lipoxygenases, and impairment of the lipid peroxide repair system, such as the cystine/glutamate antiporter (system xc-) (composed of SLC7A11 and SLC3A2) or glutathione peroxidases 4 [155, 156]. As mentioned above, HH is an iron-overload disease caused by defects in iron-sensing genes and is associated with higher cancer risk [157]. Iron overload was demonstrated to induce ferroptosis both *in vitro* and *in vivo* [158–160]. Moreover, liver damage was attenuated by the ferroptosis inhibitor ferrostatin-1 in mouse models of HH [158]. Indeed, knockout of SLC7A11 was not sufficient to trigger ferroptosis under basal iron conditions but increased the susceptibility to iron overload-elicited ferroptosis due to impaired cystine uptake and increased ROS production [158], while overexpression of SLC7A11 restored glutathione production and significantly prevented ferroptosis [160]. There is compelling evidence suggesting that there exists a tumor-suppressive nature of ferroptosis, including a metabolic link between tumor suppressors, such as p53 and BAP1, and the sensitivity of ferroptosis [161]. Recently, Wang et al. have revealed the involvement of adaptive immune responses in cancer cell ferroptosis [162]. They further demonstrated that IFN-*γ*, released from anti-PD-L1-treated activated CD8+ T cells, reduced the expression of system xc- and thus drove ferroptosis in the tumor, implying the potential efficacy of targeting iron-dependent ferroptosis in combination with cancer immunotherapy. Moreover, radiotherapy and immunotherapy act synergistically, but also independently via their respective mechanisms, to enhance tumor lipid peroxidation and ferroptosis, while ferroptosis agonists act as sensitizers for improving their antitumor efficacy [163, 164]. In fact, cisplatin, a classic chemotherapy agent, was found to induce ferroptosis through glutathione deletion in certain cancer cell lines [165]. Sorafenib is a multikinase inhibitor approved for the treatment of advanced cancer (e.g., hepatocellular carcinoma) [166], and it can be used as an agonist of ferroptosis for its inhibitory effect on system xc- [167]. More importantly, erastin, a more potent inhibitor of system xc-, has been shown to improve the anticancer activity of traditional chemotherapy drugs (e.g., docetaxel, cisplatin, and temozolomide) in several cancer cells [168–170]. Besides its promising role for cancer therapy, ferroptosis is also implicated in multiple forms of tissue damage, including ischemia/reperfusion injury [171], traumatic injury [172], and neurodegeneration [173, 174].

## 7. Conclusion and Perspective

In this review, we briefly introduce the recruitment of leukocytes into the TME and their essential roles in tumor immunosurveillance, as well as the immunosuppressive cellular components of the TME, especially TAMs and Tregs, which promote immune evasion through multiple mechanisms. Iron is critical for tumor development, and a variety of iron metabolism-related proteins are abnormally regulated in cancer, implicating dysregulated iron homeostasis as one of the metabolic hallmarks of cancer. Iron is also involved in the modulation of innate and adaptive immune responses, notably in macrophages and T cells. Therefore, targeted regulation of iron metabolism should be beneficial to antitumor immunity and cancer therapy.

Compared to traditional treatment modalities, such as chemotherapy and radiotherapy, immunotherapy presents advantages in reducing tumor recurrence and metastasis, which is based on cellular immunity. Potential treatment options targeting iron homeostasis (e.g., iron chelators, INOPs, and ferroptosis inducers), to some extent, exert their antitumor effects by enhancing antitumor immunity. The combination of immunotherapy with targeted regulation of iron and ferroptosis may be a focus of future research despite a lack of existing clinical evidence. Further studies are necessary to clarify the impact of these therapies on not only tumor cells but also other components of the TME (especially immune cells) and their states of proliferation, apoptosis, or ferroptosis. Moreover, it remains unknown whether iron deprivation for tumor suppression or iron supplementation for inducing ferroptosis is more efficient for cancer treatment. More comprehensive studies should explore the exact role of iron in regulating the crosstalk among the TME and pave the way to the development of potent iron-based therapies in the future.

## Data Availability

The availability of data and materials is not applicable.

## Abbreviations

APC: Antigen-presenting cell
CSF: Colony-stimulating factor
CTL: Cytotoxic T lymphocyte
CTLA-4: Cytotoxic T lymphocyte-associated antigen 4
CXCL: CXC-chemokine ligand
DC: Dendritic cell
DMT1: Divalent metal transporter 1
FPN: Ferroportin
FT: Ferritin
FTH: Ferritin heavy chains
FTL: Ferritin light chains
GM-CSF: Granulocyte-macrophage colony-stimulating factor
HFE: Hemochromatosis protein
HH: Hereditary hemochromatosis
HJV: Hemojuvelin
HO: Heme oxygenase
IFN: Interferon
IL: Interleukin
IONP: Iron oxide nanoparticle
LAG-3: Lymphocyte activation gene 3
LCN-2: Lipocalin-2
LIP: Labile iron pool
MHC: Major histocompatibility complex
NK: Natural killer
PD-1: Programmed cell death protein 1
PD-L1: Programmed cell death-ligand 1
ROS: Reactive oxygen species
STEAP: Six-transmembrane epithelial antigen of the prostate
TAM: Tumor-associated macrophage
TBI: Transferrin-bound iron
TF: Transferrin
TFR: Transferrin receptor
TGF: Transforming growth factor
Th: Helper T cell
TIM-3: T cell immunoglobulin and mucin domain 3
TME: Tumor microenvironment
TNF: Tumor necrosis factor
Treg: T regulatory cell
VISTA: V domain Ig suppressor of T cell activation
ZIP: Zrt/Irt-like protein.
